# Supplementary material for: Foodborne Event Detection Based on Social Media Mining: A Systematic Review
Source: Foods. 2025 Jan 14;14(2):239. doi: 10.3390/foods14020239 (PMC11764790; doi:10.3390/foods14020239)
Supplement: Supplementary file 1 [file foods-14-00239-s001.zip › foods-3353209-supplementary.pdf]

Figure S1 PRISMA Flowchart

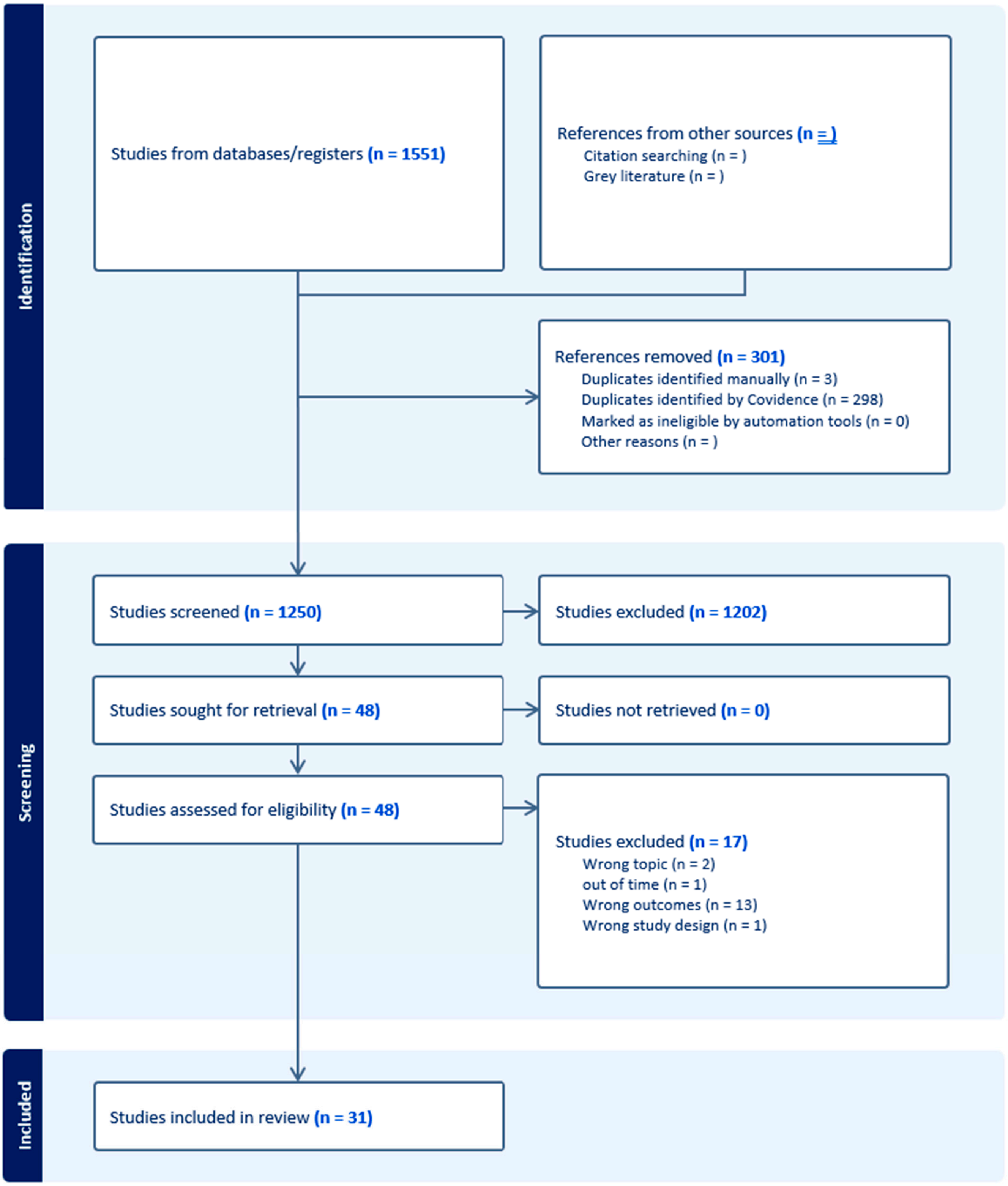

Table S1 Search strategy for PubMed, last run 15 December 2024

("social-media"[MeSH Terms] OR “social-media”[Text Word]<br>OR "social-networking"[MeSH Terms] OR “social-network”[Text Word]<br>OR "social networking"[MeSH Terms] OR “social network”[Text Word]<br>OR “social networks”[Text Word]<br>OR “Twitter”[Text Word]<br>OR “WhatsApp”[Text Word]<br>OR “Facebook”[Text Word]<br>OR “twitch”[Text Word]<br>OR “Weibo”[Text Word]<br>OR “Snapchat”[Text Word]<br>OR TikTok[Text Word]<br>OR “YouTube”[Text Word]<br>OR “Instagram”[Text Word]<br>OR “tripadvisor”[Text Word]<br>OR “reddit” [Text Word]<br>OR “MySpace”[Text Word]<br>OR “Orkut”[Text Word]<br>OR “WeChat”[Text Word]<br>OR “Google+”[Text Word]<br>OR “blog”[Text Word]<br>OR “Neverblog”[Text Word]<br>OR “QQ”[Text Word]<br>OR qzone[Text Word]<br>OR “Viber”[Text Word])<br>AND<br>("Data-Mining"[Mesh]<br>OR "sentiment-analysis"[All fields]<br>OR "Data-Mining"[All fields]<br>OR "Text-Mining"[All fields]<br>OR "machine-learning"[MeSH Terms]<br>OR “machine-learning”[Text Word]<br>OR “deep learning”[Text Word]<br>OR "neural networks, computer"[Text Word]

OR "natural language processing"[MeSH Terms]
OR natural language processing[Text Word]
OR "artificial intelligence"[MeSH Terms]
OR “ai artificial intelligence”[Text Word]
OR “artificial intelligence”[Text Word])
AND
("Foodborne-Diseases"[Mesh]
OR "Foodborne" [All Fields]
OR "Foodborne-illness" [All Fields]
OR "Dietary-Exposure" [Mesh]
OR "Population-Surveillance"[Mesh]
OR "Dietary-Exposure"[All fields]
OR “Foodborne disease outbreaks” [Title/Abstract]
OR "disease outbreaks"[MeSH Terms]
OR “disease outbreak”[Text Word]
OR "Bacillus licheniformis"[Mesh]
OR "Bacillus licheniformis"[Title/Abstract]
OR "Botulism"[Mesh]
OR "Botulism"[Title/Abstract]
OR "Cronobacter"[Mesh]
OR "Cronobacter"[Title/Abstract]
OR "bacillus licheniformis"[MeSH Terms]
OR “Bacillus licheniformis”[Title/Abstract]
OR "cronobacter"[MeSH Terms]
OR cronobacter[Title/Abstract]
OR "norovirus"[MeSH Terms]
OR Norovirus[Title/Abstract]
OR "salmonella"[MeSH Terms]
OR Salmonella[Title/Abstract]
OR salmonellosis[Title/Abstract]
OR "clostridium perfringens"[MeSH Terms]
OR “Clostridium perfringens” [Title/Abstract]
OR "campylobacter"[MeSH Terms]
OR Campylobacter[Title/Abstract]
OR "staphylococcus aureus"[MeSH Terms]

OR Staphylococcus aureus[Title/Abstract]
OR "clostridium botulinum"[MeSH Terms]
OR “Clostridium botulinum”[Title/Abstract]
OR "listeria"[MeSH Terms]
OR Listeria[Title/Abstract]
OR "escherichia coli"[MeSH Terms]
OR “Escherichia coli”[Title/Abstract]
OR "vibrio"[MeSH Terms]
OR Vibrio[Title/Abstract]
OR "gastrointestinal microbiome"[MeSH Terms]
OR “enteric bacteria”[Text Word])

Table S2 Risk of Bias evaluation for each domain using PROBAST scale

	Domain 1: Participants				Domain 2: Predictors						Domain 3: Outcome												Domain 4: Analysis													
	**1 MD**	**1 MV**	**2 MD**	**2 MV**	**3 MD**	**3 MV**	**4 MD**	**4 MV**	**5 MD**	**5 MV**	**6 MD**	**6 MV**	**7 MD**	**7 MV**	**8 MD**	**8 MV**	**9 MD**	**9 MV**	**10 MD**	**10 MV**	**11 MD**	**11 MV**	**12 MD**	**12 MV**	**13 MD**	**13 MV**	**14 MD**	**14 MV**	**15 MD**	**15 MV**	**16 MD**	**16 MV**	**17 MD**	**17 MV**	**18 MD**	**18 MV**
Cui et al. 2017 [1]	NI	NI	NI	NI	PY	PY	NI	NI	PY	PY	NI	NI	NI	NI	NI	NI	PY	PY	NI	NI	NI	NI	NI	NI	NI	NI	NI	NI	NI	NI	NI	NI	NI	NI	NI	NI
Denecke et al. 2013 [2]	NI	NI	PN	PN	PY	PY	PN	PN	PY	PY	PY	PY	NI	NI	PN	PN	PN	PN	NI	NI	NI	NI	NI	NI	NI	NI	NI	NI	NI	NI	PY	PY	NI	NI	NI	NI
Effland et al. 2018 [3]	NI	NI	PN	PN	PY	PY	PN	PN	PY	PY	PY	PY	NI	NI	PN	PN	PN	PN	NI	NI	NI	NI	NI	NI	NI	NI	NI	NI	PY	PY	PY	PY	NI	NI	NI	NI
Erraguntla et al. 2019 [4]	NI	NI	PN	PN	PY	PY	PN	PN	PY	PY	PY	PY	PY	PY	PN	PN	PN	PN	PY	PY	NI	NI	PY	PY	PY	PY	NI	NI	PY	PY	PY	PY	NI	NI	NI	NI
Gao et al. 2021 [5]	NI	NI	PN	PN	PY	PY	PN	PN	PY	PY	PY	PY	PY	PY	PN	PN	PN	PN	NI	NI	NI	NI	NI	NI	NI	NI	NI	NI	NI	NI	PY	PY	PY	PY	NI	NI
Glowacki et al 2019 [6]	NI	NI	NI	NI	PY	PY	NI	NI	PY	PY	NI	NI	NI	NI	NI	NI	PY	PY	NI	NI	NI	NI	NI	NI	NI	NI	NI	NI	NI	NI	NI	NI	NI	NI	NI	NI
Harris et al. 2014 [7]	NI	NI	NI	NI	NI	NI	NI	NI	NI	NI	NI	NI	NI	NI	NI	NI	NI	NI	NI	NI	NI	NI	NI	NI	NI	NI	NI	NI	NI	NI	NI	NI	NI	NI	NI	NI
Harris et al. 2017 [8]	PN	PN	NI	NI	PY	PY	PN	PN	PY	PY	NI	NI	NI	NI	PN	PN	PY	PY	NI	NI	NI	NI	NI	NI	NI	NI	NI	NI	NI	NI	PY	PY	NI	NI	NI	NI
Harrison et al. 2014 [9]	NI	NI	PY	PY	PY	PY	PY	PY	PY	PY	PY	PY	PY	PY	PY	PY	PY	PY	NI	NI	NI	NI	NI	NI	NI	NI	NI	NI	NI	NI	NI	NI	NI	NI	NI	NI
Hu et al. 2022 [10]	NI	NI	PN	PN	PY	PY	PN	PN	PY	PY	PY	PY	PY	PY	PN	PN	PN	PN	NI	NI	NI	NI	NI	NI	PY	PY	NI	NI	NI	NI	PY	PY	NI	NI	NI	NI
Hu et al.2023 [11]	PY	PY	PY	PY	NI	NI	NI	NI	NI	NI	PY	PY	PY	PY	PN	PN	PN	PN	PY	PY	PY	PY	PY	PY	NI	NI	PY	PY	PY	PY	PY	PY	PY	PY	NI	NI
Joaristi et al. 2016 [12]	NI	NI	PN	PN	PY	PY	PN	PN	PY	PY	NI	NI	NI	NI	PN	PN	PN	PN	NI	NI	NI	NI	NI	NI	PN	PN	NI	NI	NI	NI	PY	PY	NI	NI	NI	NI

Kate et al. 2014[13]	NI	NI	PN	PN	PY	PY	PN	PN	PY	PY	PY	PY	PY	PY	PN	PN	PN	PN	NI	NI	NI	NI	PY	PY	NI	NI	NI	NI	PY	PY	PY	PY	NI	NI	NI	NI
Lee et al. 2023 [14]	Y	NI	Y	NI	Y	NI	Y	NI	Y	NI	Y	NI	Y	NI	Y	NI	Y	NI	Y	NI	Y	NI	Y	NI	Y	NI	N	NI	Y	NI	Y	NI	Y	NI	NI	NI
Maharana et al. 2019 [15]	NI	NI	PN	PN	PY	PY	PN	PN	PY	PY	PY	PY	PY	PY	PN	PN	PN	PN	NI	NI	NI	NI	NI	NI	PN	PN	NI	NI	NI	NI	PY	PY	NI	NI	NI	NI
Mejia et al.2019 [16]	NI	NI	PN	PN	PY	PY	PN	PN	PY	PY	PY	PY	PY	PY	PN	PN	PN	PN	NI	NI	NI	NI	NI	NI	PY	PY	NI	NI	NI	NI	PY	PY	NI	NI	NI	NI
Molenaar et al. 2024 [17]	PY	NI	PY	NI	PY	NI	PY	NI	PY	NI	PY	NI	Y	PY	PY	NI	PY	NI	PY	NI	Y	Y	PY	NI	NI	NI	PY	NI	N	N	PY	NI	N	N	NI	NI
Nsoesie et al. 2014 [18]	NI	NI	PN	PN	PY	PY	PN	PN	PY	PY	PY	PY	PY	PY	PN	PN	PN	PN	PN	PN	NI	NI	NI	NI	PN	PN	NI	NI	NI	NI	PY	PY	NI	NI	NI	NI
Rizzoli et al. 2021 [19]	NI	NI	PN	PN	PY	PY	PN	PN	PY	PY	PY	PY	PY	PY	PN	PN	PN	PN	NI	NI	NI	NI	NI	NI	PN	PN	NI	NI	NI	NI	NI	NI	PY	PY	NI	NI
Sadilek et al. [20]	NI	NI	NI	NI	PY	PY	PN	PN	PY	PY	NI	NI	NI	NI	PN	PN	PN	PN	NI	NI	NI	NI	NI	NI	NI	NI	NI	NI	NI	NI	NI	NI	NI	NI	NI	NI
Sadilek et al. 2018 [21]	NI	NI	NI	NI	PY	PY	PN	PN	PY	PY	NI	NI	NI	NI	PN	PN	PN	PN	NI	NI	NI	NI	NI	NI	NI	NI	NI	NI	NI	NI	NI	NI	NI	NI	NI	NI
Schomberg et al. 2016 [22]	PY	PY	NI	NI	PY	PY	NI	NI	PY	PY	PY	PY	PY	PY	NI	NI	NI	NI	NI	NI	NI	NI	NI	NI	NI	NI	NI	NI	NI	NI	PY	PY	PY	PY	NI	NI
Serban et al. 2019 [23]	NI	NI	PN	PN	PY	PY	PN	PN	PY	PY	PY	PY	NI	NI	PN	PN	PN	PN	NI	NI	PY	PY	NI	NI	PN	PN	NI	NI	NI	NI	PY	PY	NI	NI	NI	NI
Tao et al. 2021 [24]	NI	NI	PN	PN	PY	PY	PN	PN	PY	PY	PY	PY	NI	NI	PN	PN	PN	PN	NI	NI	NI	NI	NI	NI	PN	PN	NI	NI	NI	NI	PY	PY	NI	NI	NI	NI
Tao et al. 2023 [25]	PY	NI	PY	PY	PY	NI	PY	NI	PY	NI	PY	NI	PY	NI	PN	NI	PN	NI	PY	NI	PY	NI	NI	NI	NI	NI	NI	NI	NI	NI	NI	NI	NI	NI	NI	NI
Tegtmeyer et al. 2012 [26]	NI	NI	NI	NI	NI	NI	NI	NI	NI	NI	NI	NI	NI	NI	NI	NI	NI	NI	NI	NI	NI	NI	NI	NI	NI	NI	NI	NI	NI	NI	NI	NI	NI	NI	NI	NI
Vasanthakumar	PY	PY	PY	PY	PY	PY	PN	PY	PY	PY	PY	PY	NI	NI	PY	PY	PN	PY	PY	PY	PY	PY	PY	PY	NI	NI	PY	PY	PY	PY	PY	PY	PY	PY	PY	PY

et al. 2023[27]																																				
Wang et al. 2017 [28]	NI	NI	NI	NI	PY	PY	PN	PN	PY	PY	NI	NI	PN	PN	PN	PN	PY	PY	NI	NI	NI	NI	NI	NI	NI	NI	NI	NI	NI	NI	PY	PY	NI	NI	NI	NI
Widener et al. 2014 [29]	NI	NI	PN	PN	PY	PY	PN	PN	PY	PY	PY	PY	NI	NI	PN	PN	PN	PN	NI	NI	NI	NI	NI	NI	PN	PN	NI	NI	NI	NI	NI	NI	NI	NI	NI	NI
Zhang et al. 2019 [30]	NI	NI	NI	NI	PY	PY	PN	PN	PY	PY	NI	NI	PY	PY	PN	PN	PN	PN	NI	NI	NI	NI	NI	NI	NI	NI	NI	NI	NI	NI	NI	NI	NI	NI	NI	NI
Zou et al. 2016 [31]	PY	PY	NI	NI	PY	PY	PN	PN	PY	PY	PY	PY	PY	PY	PN	PN	PN	PN	PY	PY	NI	NI	PY	PY	NI	NI	NI	NI	NI	NI	PY	PY	PY	PY	NI	NI

Participants: 1) Were appropriate data sources used, e.g. cohort, RCT or nested case-control study data?; 2) Were all inclusions and exclusions of participants appropriate? Predictors: 3) Were predictors defined and assessed in a similar way for all participants?; 4) Were predictor assessments made without knowledge of outcome data? 5) Are all predictors available at the time the model is intended to be used? Outcome: 6) Was the outcome determined appropriately? 7) Was a pre-specified or standard outcome definition used? 8) Were predictors excluded from the outcome definition? 9) Was the outcome determined without knowledge of predictor information? 10) Was the time interval between predictor assessment and outcome determination appropriate? Analysis: 11) Were there a reasonable number of participants with the outcome? 12) Were continuous and categorical predictors handled appropriately? 13) Were participants with missing data handled appropriately? 14) Was selection of predictors based on univariable analysis avoided? 15) Were complexities in the data (e.g. censoring, competing risks, sampling of controls) accounted for appropriately? 16) Were relevant model performance measures evaluated appropriately? 17) Were model overfitting and optimism in model performance accounted for? 18) Do predictors and their assigned weights in the final model correspond to the results from multivariable analysis? Abbreviations: MD, model development; MV, Model Validation

References

1. Cui, W.; Wang, P.; Du, Y.; Chen, X.; Guo, D.; Li, J.; Zhou, Y. An Algorithm for Event Detection Based on Social Media Data. *Neurocomputing* **2017**, *254*, 53–58, doi:10.1016/j.neucom.2016.09.127.
2. Denecke, K.; Krieck, M.; Otrusina, L.; Smrz, P.; Dolog, P.; Nejdl, W.; Velasco, E. How to Exploit Twitter for Public Health Monitoring? *Methods Inf Med* **2013**, *52*, 326–339, doi:10.3414/ME12-02-0010.
3. Effland, T.; Lawson, A.; Balter, S.; Devinney, K.; Reddy, V.; Waechter, H.; Gravano, L.; Hsu, D. Discovering Foodborne Illness in Online Restaurant Reviews. *Journal of the American Medical Informatics Association* **2018**, *25*, 1586–1592, doi:10.1093/jamia/ocx093.
4. Erraguntla, M.; Zapletal, J.; Lawley, M. Framework for Infectious Disease Analysis: A Comprehensive and Integrative Multi-Modeling Approach to Disease Prediction and Management. *Health Informatics J* **2019**, *25*, 1170–1187, doi:10.1177/1460458217747112.
5. Gao, W.; Fang, Y.; Li, L.; Tao, X. Event Detection in Social Media via Graph Neural Network. In Proceedings of the Web Information Systems Engineering – WISE 2021; Zhang, W., Zou, L., Maamar, Z., Chen, L., Eds.; Springer International Publishing: Cham, 2021; pp. 370–384.
6. Glowacki, E.M.; Glowacki, J.B.; Chung, A.D.; Wilcox, G.B. Reactions to Foodborne Escherichia Coli Outbreaks: A Text-Mining Analysis of the Public's Response. *American Journal of Infection Control* **2019**, *47*, 1280–1282, doi:10.1016/j.ajic.2019.04.004.
7. Harris, J.K.; Mansour, R.; Choucair, B.; Olson, J.; Nissen, C.; Bhatt, J. Health Department Use of Social Media to Identify Foodborne Illness — Chicago, Illinois, 2013–2014. *MMWR Morb Mortal Wkly Rep* **2014**, *63*, 681–685.
8. Harris, J.K.; Hawkins, J.B.; Nguyen, L.; Nsoesie, E.O.; Tuli, G.; Mansour, R.; Brownstein, J.S. Using Twitter to Identify and Respond to Food Poisoning: The Food Safety STL Project. *J Public Health Manag Pract* **2017**, *23*, 577–580, doi:10.1097/PHH.0000000000000516.
9. Harrison, C.; Jorder, M.; Stern, H.; Stavinsky, F.; Reddy, V.; Hanson, H.; Waechter, H.; Lowe, L.; Gravano, L.; Balter, S. Using Online Reviews by Restaurant Patrons to Identify Unreported Cases of Foodborne Illness — New York City, 2012–2013. *MMWR Morb Mortal Wkly Rep* **2014**, *63*, 441–445.
10. Hu, R.; Zhang, D.; Tao, D.; Hartvigsen, T.; Feng, H.; Rundensteiner, E. TWEET-FID: An Annotated Dataset for Multiple Foodborne Illness Detection Tasks 2022.
11. Hu, R.; Zhang, D.; Tao, D.; Zhang, H.; Feng, H.; Rundensteiner, E. UCE-FID: Using Large Unlabeled, Medium Crowdsourced-Labeled, and Small Expert-Labeled Tweets for Foodborne Illness Detection 2023.
12. Joaristi, M.; Serra, E.; Spezzano, F. Evaluating the Impact of Social Media in Detecting Health-Violating Restaurants. In Proceedings of the 2016 IEEE/ACM International Conference on Advances in Social Networks Analysis and Mining (ASONAM); August 2016; pp. 626–633.
13. Kate, K.; Negi, S.; Kalagnanam, J. Monitoring Food Safety Violation Reports from Internet Forums. In *e-Health – For Continuity of Care*; IOS Press, 2014; pp. 1090–1094.
14. Lee, C.K.H. Predicting Food Safety Violations via Social Media to Improve Public Health Surveillance. *ECSM* **2023**, *10*, 109–116, doi:10.34190/ecsm.10.1.1009.
15. Maharana, A.; Cai, K.; Hellerstein, J.; Hswen, Y.; Munsell, M.; Staneva, V.; Verma, M.; Vint, C.; Wijaya, D.; Nsoesie, E.O. Detecting Reports of Unsafe Foods in Consumer Product Reviews. *JAMIA Open* **2019**, *2*, 330–338, doi:10.1093/jamiaopen/ooz030.

16. Mejia, J.; Mankad, S.; Gopal, A. A for Effort? Using the Crowd to Identify Moral Hazard in New York City Restaurant Hygiene Inspections. *Information Systems Research* **2019**, *30*, 1363–1386, doi:10.1287/isre.2019.0866.
17. Molenaar, A.; Lukose, D.; Brennan, L.; Jenkins, E.L.; McCaffrey, T.A. Using Natural Language Processing to Explore Social Media Opinions on Food Security: Sentiment Analysis and Topic Modeling Study. *Journal of Medical Internet Research* **2024**, *26*, e47826, doi:10.2196/47826.
18. Nsoesie, E.O.; Kluberg, S.A.; Brownstein, J.S. Online Reports of Foodborne Illness Capture Foods Implicated in Official Foodborne Outbreak Reports. *Preventive Medicine* **2014**, *67*, 264–269, doi:10.1016/j.ypmed.2014.08.003.
19. Rizzoli, V.; Mascarello, G.; Pinto, A.; Crovato, S.; Ruzza, M.; Tiozzo, B.; Ravarotto, L. 'Don't Worry, Honey: It's Cooked': Addressing Food Risk during Pregnancy on Facebook Italian Posts. *Foods* **2021**, *10*, 2484, doi:10.3390/foods10102484.
20. Sadilek, A.; Kautz, H.; DiPrete, L.; Labus, B.; Portman, E.; Teitel, J.; Silenzio, V. Deploying nEmesis: Preventing Foodborne Illness by Data Mining Social Media. *AI Magazine* **2017**, *38*, 37–48, doi:10.1609/aimag.v38i1.2711.
21. Sadilek, A.; Caty, S.; DiPrete, L.; Mansour, R.; Schenk, T.; Bergtholdt, M.; Jha, A.; Ramaswami, P.; Gabrilovich, E. Machine-Learned Epidemiology: Real-Time Detection of Foodborne Illness at Scale. *npj Digital Med* **2018**, *1*, 1–7, doi:10.1038/s41746-018-0045-1.
22. Schomberg, J.P.; Haimson, O.L.; Hayes, G.R.; Anton-Culver, H. Supplementing Public Health Inspection via Social Media. *PLOS ONE* **2016**, *11*, e0152117, doi:10.1371/journal.pone.0152117.
23. Șerban, O.; Thapen, N.; Maginnis, B.; Hankin, C.; Foot, V. Real-Time Processing of Social Media with SENTINEL: A Syndromic Surveillance System Incorporating Deep Learning for Health Classification. *Information Processing & Management* **2019**, *56*, 1166–1184, doi:10.1016/j.ipm.2018.04.011.
24. Tao, D.; Zhang, D.; Hu, R.; Rundensteiner, E.; Feng, H. Crowdsourcing and Machine Learning Approaches for Extracting Entities Indicating Potential Foodborne Outbreaks from Social Media. *Sci Rep* **2021**, *11*, 21678, doi:10.1038/s41598-021-00766-w.
25. Tao, D.; Hu, R.; Zhang, D.; Laber, J.; Lapsley, A.; Kwan, T.; Rathke, L.; Rundensteiner, E.; Feng, H. A Novel Foodborne Illness Detection and Web Application Tool Based on Social Media. *Foods* **2023**, *12*, 2769, doi:10.3390/foods12142769.
26. Tegtmeyer, R.; Potts, L.; Hart-Davidson, W. Tracing and Responding to Foodborne Illness. In Proceedings of the Proceedings of the 30th ACM international conference on Design of communication; Association for Computing Machinery: New York, NY, USA, October 3 2012; pp. 369–370.
27. Vasanthakumar, U.; Goh, J.R.B.; Hui, S.C.; Lam, K.Y.; Er, B.; Fua'di, M.T.; Aung, K.T. Fine-Tuning Pre-Trained Language Model for Urgency Classification on Food Safety Feedback. In Proceedings of the 2023 10th International Conference on ICT for Smart Society (ICISS); IEEE, 2023; pp. 01–10.
28. Wang, Z.; Balasubramani, B.S.; Cruz, I.F. Predictive Analytics Using Text Classification for Restaurant Inspections. In Proceedings of the Proceedings of the 3rd ACM SIGSPATIAL Workshop on Smart Cities and Urban Analytics; Association for Computing Machinery: New York, NY, USA, November 7 2017; pp. 1–4.
29. Widener, M.J.; Li, W. Using Geolocated Twitter Data to Monitor the Prevalence of Healthy and Unhealthy Food References across the US. *Applied Geography* **2014**, *54*, 189–197, doi:10.1016/j.apgeog.2014.07.017.

30. Zhang, M.; Guo, D.; Hu, J.; Jin, W. Risk Prediction and Assessment of Foodborne Disease Based on Big Data. In Proceedings of the Proceedings of the 5th ACM SIGSPATIAL International Workshop on the Use of GIS in Emergency Management; Association for Computing Machinery: New York, NY, USA, July 30 2020; pp. 1–6.
31. Zou, B.; Lampos, V.; Gorton, R.; Cox, I.J. On Infectious Intestinal Disease Surveillance Using Social Media Content. In Proceedings of the Proceedings of the 6th International Conference on Digital Health Conference; Association for Computing Machinery: New York, NY, USA, April 11 2016; pp. 157–161.
